# Isolation by Miniaturized Culture Chip of an Antarctic bacterium *Aequorivita* sp. with antimicrobial and anthelmintic activity

**DOI:** 10.1016/j.btre.2018.e00281

**Published:** 2018-09-06

**Authors:** Fortunato Palma Esposito, Colin J. Ingham, Raquel Hurtado-Ortiz, Chantal Bizet, Deniz Tasdemir, Donatella de Pascale

**Affiliations:** a Institute of Protein Biochemistry, National Research Council, Naples, 80131, Italy; b Hoekmine BV, Utrecht, 3584 CS, The Netherlands; c CIP-Collection of Institut Pasteur, Department of Microbiology, Institut Pasteur, Paris, 75015, France; d CRBIP-Biological Resource Centre, Department of Microbiology, Institut Pasteur, Paris, 75015, France; e GEOMAR Centre for Marine Biotechnology (GEOMAR-Biotech), Research Unit Marine Natural Products Chemistry, GEOMAR Helmholtz Centre for Ocean Research Kiel, Kiel, 24106, Germany; f Marine Biotechnology Department, Stazione Zoologica Anton Dohrn, Villa Comunale, Naples, 80121, Italy

**Keywords:** *Aequorivita*, Antarctic bacteria, Miniaturized culture chip, Multidrug resistant bacteria, Genome mining, Antimicrobial, Anthelmintic

## Abstract

Microbes are prolific sources of bioactive molecules; however, the cultivability issue has severely hampered access to microbial diversity. Novel secondary metabolites from as-yet-unknown or atypical microorganisms from extreme environments have realistic potential to lead to new drugs with benefits for human health. Here, we used a novel approach that mimics the natural environment by using a Miniaturized Culture Chip allowing the isolation of several bacterial strains from Antarctic shallow water sediments under near natural conditions. A Gram-negative Antarctic bacterium belonging to the genus *Aequorivita* was subjected to further analyses. The *Aequorivita* sp. genome was sequenced and a bioinformatic approach was applied to identify biosynthetic gene clusters. The extract of the *Aequorivita* sp. showed antimicrobial and anthelmintic activity towards Multidrug resistant bacteria and the nematode *Caenorhabditis elegans*. This is the first multi-approach study exploring the genomics and biotechnological potential of the genus *Aequorivita* that is a promising candidate for pharmaceutical applications.

## Highlights

• Novel microbial isolation approach allowed the identification of a Gram-negative Antarctic bacterium belonging to the genus *Aequorivita*.
• *Aequorivita* sp. showed antimicrobial and anthelmintic activity without toxic effect towards eukaryotic cells.
• The whole genome of *Aequorivita* sp. was sequenced and compared with other strains to identify biosynthetic gene clusters.
• This novel approach represents a promising strategy to isolate rare or novel strains useful for biotechnological applications.

## 1 Introduction

The total number of bacterial species in the world is estimated to vary from 10^7^ to 10^9^ [1], but most of them have never been cultivated. Culture-independent methods have demonstrated the enormous richness of biodiversity in the microbial world [2,3], but this wealth remains largely inaccessible. The “challenge of uncultivable microorganisms” is a topic that has been concerning scientists for many years. Solutions to this problem could facilitate the discovery of new strains holding novel and different features, which biotechnology could exploit. The problem, which is also known as the *“great plate count anomaly”* [4,5] is based on the observation, that less than 1% of microbes present in an environmental sample can be grown on an agar plate. Although there is a large number of reasons that marine bacteria cannot be easily cultured in laboratory conditions, the problem can often be the difficulty of replicating the very precise environmental conditions required for the growth. Free-living microbes can have a broad array of environmental requirements, for example many organisms cannot survive in high-nutrient conditions common to many types of culture media. The development of low-nutrient media has greatly increased the number of organisms that have been successfully cultured [6]. An even more serious problem is that many marine microorganisms have adapted to their low resource environment by growing very slowly or entering in a dormant stage, hence it is necessary to find the right conditions to trigger entry into cell replication [6]. Among the strategies for the isolation and cultivation of new or atypical microorganisms, the simulation of the natural environment is probably the most promising. The use of innovative devices that are able to mimic natural conditions enhances the possibility of cultivating different microbial species. One of these systems is the Miniaturized Culture Chip (MCC), which is a disposable array of thousands of miniaturized Petri dish on a chip [7]. One of the major advantages of the MCC is that it can be placed directly on natural samples (such as sediments) with nutrients available to the microorganisms via a porous ceramic that forms the base. This device simulates the natural habitat, in which microorganisms live in community, and allows the communication among microorganisms [[7], [8], [9]]. The isolation of new strains also enables exploration of the chemical diversity of natural products they synthesize. Microorganisms are well-known for producing a large variety of antimicrobial agents. Marine microorganisms are no exception and they have also proven to be a rich source of potent natural products with antimicrobial, antiinflammatory, antiviral, and anticancer activity [10]. In particular, the discovery of novel secondary metabolites as antibiotic lead compounds is urgently needed in order to counteract the spread of MultiDrug Resistant (MDR) bacteria. Recently, the World Health Organization [11] published its first ever list of antibiotic-resistant "priority pathogens" – a catalogue of 12 families of bacteria that pose the greatest threat to human health. The list includes MDR bacteria such as *Acinetobacter baumannii, Pseudomonas aeruginosa* and methicillin-resistant *Staphylococcus aureus* (MRSA). They are able to provoke recalcitrant infectious, especially in hospitals where they pose the major challenge to patient safety, resulting in one of the leading causes of death in the USA and Europe with high costs for their public health [12]. The redundancy in the discovery of already known compounds, generally produced by well-known and repeatedly isolated species, increases the demand of searching for novel drugs. In the search of new antibiotics or new molecules of pharmaceutical interest, the exploration of microorganisms from extreme environments may lead to the identification of strains that can provide novel types of compounds for biotechnology [13]. Antarctica is one of the most extreme places on Earth. The isolated and unique Nature of Antarctica, characterized by low temperatures, oligotrophic environment, long periods of light/dark, has drawn the attention of the scientists. Antarctica and the surrounding oceans represent an untapped area for exploring biodiversity and potentially unknown organisms adapted to the extreme living conditions. These extremophiles exhibit physical and chemical adaptations not found elsewhere on the planet [14]. The genus *Aequorivita* (family Flavobacteriaceae) was discovered for the first time in 2002 from Antarctic terrestrial and marine habitats [15]. The members of this genus are Gram-negative, non-motile, psychrotolerant, strictly aerobic bacteria, producing orange or yellow pigments. Thus far, the genus *Aequorivita* has remained rarely isolated and poorly investigated. Previous studies have reported the taxonomy, fatty acid composition and DNA G + C content of *A. lipolytica*, *A. viscosa*, *A. sublithincola*, *A. crocea*, *A. antarctica* and *A. capsosiphonis* [15]. Herein we report the isolation of a poorly investigated strain of *Aequorivita* sp., from Antarctic shallow water sediments by employing the MCC method, as well as its whole genome sequencing, the analysis of the biosynthetic gene clusters and the evaluation of its antimicrobial and anthelmintic potential. To our knowledge, this is the first study exploring the genus *Aequorivita* for its bioactivity.

## 2 Material and Methods

### 2.1 Collection of Antarctic sediments

Shallow water (50 cm depth) sediments were collected by using sterile 50 mL Falcon tubes, (Sarstedt) in January 2014, from 3 different sites during an expedition in the framework of National Program for Antarctic Research of Italy (PNRA) in the area of Edmonson Point, Antarctica, 74° 20′ (74.3333°) South, 165° 8′ (165.1333°) East. The samples were transported to the laboratory in dry ice and stored at −80 °C until initializing the experiments.

### 2.2 Design, fabrication and preparation of culture chips for the isolation of microorganisms

**Fig. 1:**
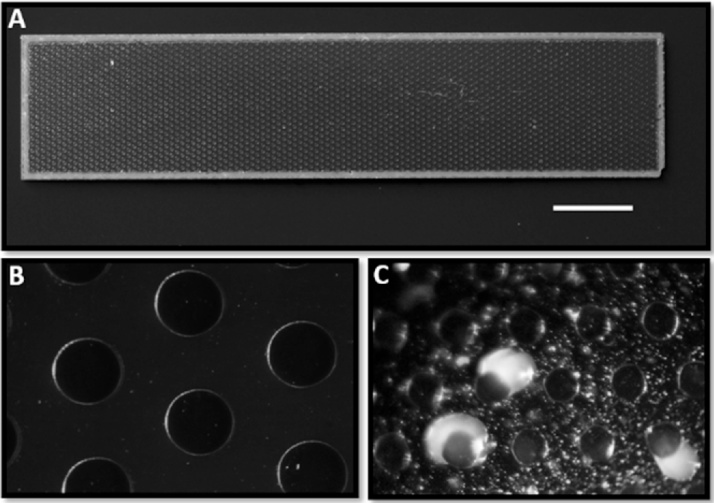
MCC180.10 culture chip picture (A). Microscope view of the MCC180.10 (B). Bacterial colonies visible on the chip after 7 days of incubation (C). The scale bar in (A) indicates 4 mm when applied to (A) 200 μm when applied to (B) and 360 μm when applied to (C).

MCC was used to isolate microorganisms grown as microcolonies [9]. MCC contained arrays of microwells with a Porous Aluminum Oxide (PAO) base (8 × 36 mm, 60 μm thick, 40% porosity, 20 to 200 μm pore diameter) acting as a sterile filter. MCC was fabricated by patterning the wall material (Ordyl 300 film, Elga, Italy) using photolithography and then applying it to 8 × 36 mm strips of PAO arrayed on a silicon wafer. Walls were patterned by photolithography of 10 μm thick Ordyl 300 film according to the manufacturer’s protocols. The resulting perforated and processed material was heat/pressure applied to the PAO. Platinum (10 nm) was used to sputter coat the upper surface of the MCC. Wells had 180 μm diameter, spaced 160 μm in a hexagonal patterning with 4500 microwells per chip. MCC was sterilized using a high-intensity UV ozone cleaner (PSD, Novascan, USA) for 15 min. It is basically a high number of miniaturized Petri dishes on a chip containing wells which can host one or more microbes. On the bottom of the chip there is a filter of 0.22 μm which circumvents the passage of the microbes through the chip. So, it can be placed directly on sediments or on a flat surface and inoculated with the sample [7]. For this experiment, two different growth conditions were used in order to evaluate the effect of different nutrients on the microbial growth. Condition A, minimal: sediment was packed into a Petri dish and overlaid with a thin layer of agarose 1% (w/v) mixed with a filtered solution of 0.001% (w/v) FeSO_4_ · 7H_2_O. Condition B, richer: sediment was packed into a Petri dish and overlaid with a thin layer of agarose 1% (w/v) mixed with a filtered solution of 0.001% (w/v) FeSO4 · 7H2O, 3% (w/v) Sea Salt, 1 g/L Peptone, 0.5 g/L Yeast Extract. Sterile cultivation chips were placed on the solidified mixture composed by sediments, nutrients and agarose. Then, 0.5 g of Antarctic sediments (same sediments within the chip) were dissolved in 1 mL of sterile water, shaken by using a vortex and subjected to 10-fold serial dilutions until the dilution 10^−5^. Three μL of each dilution was gently spread-plated on the chip by using a 1 μL sterile plastic loop. Finally, not all the wells will contain microorganisms. After 10 and 45 days of incubation at 7 °C the recovery of microcolonies from wells was carried out using a fine sterile toothpick using a dissection microscope to visualize the target microcolony (see Fig. 1). Finally, picked microcolonies were dissolved in sterile water. Half of the sample was used to perform the identification, while the other half was stored at −80 °C with 20% (v/v) glycerol.

### 2.3 Molecular identification and phylogenetic analysis of isolated strains

The phylogenetic affiliation of bacterial isolates was performed through the 16S rRNA genes amplification and analysis. The freeze and thaw method was used to obtain bacterial genomic DNA. In particular, a colony of each isolate was picked, dissolved in 100 μL of sterile water, transferred in 0.5 mL Eppendorf-tube, mixed with a shaker and frozen at -20 °C for minimum 1 h. Thereafter, the frozen tubes were incubated for 15 min at 99 °C in a thermocycler, centrifuged for 10 min at 8900 rpm and then 50 μL of supernatant was transferred in a new 0.5 mL Eppendorf-tube and used as template for the amplification via PCR of 16S rDNA genes. PCR was carried out in a total volume of 50 μL containing DreamTaq PCR Master Mix (a ready-to-use solution containing DreamTaq DNA Polymerase, optimized DreamTaq buffer, MgCl_2_, and dNTPs) and 1 μM of primer Eub27 F (Forward, seq: 5′-AGAGTTTGATCCTGGCTCAG-3′) and Univ1492R (Reverse, seq: 5′-GGTTACCTTGTTACGACTT-3′) [16]. The reaction conditions used were: one cycle (93 °C for 2 min), 30 cycles (92 °C for 30 s, 55 °C for 30 s, and 72 °C for 1 min), with a final extension of 5 min at 72 °C. PCR products were then purified and sequenced by Institut für Klinische Molekularbiologie (IKMB), Kiel University, and submitted to BLAST for the phylogenetic affiliation. The strain identified as *Aequorivita* sp. was selected for further studies and the 16S rRNA gene sequence was deposited in GenBank (accession number MH012204). MEGA7 software [17] was used to align the sequences and to construct the phylogenetic tree. The evolutionary history was inferred using the Neighbor-Joining method.

### 2.4 Genome sequencing of *Aequorivita* sp.

The whole genome of *Aequorivita* sp., and other two *Aequorivita* strains (stored at Biological Resource Center of Institut Pasteur) *A. lipolytica* CIP 107455^T^, and *A. antarctica* CIP 107457^T^ was obtained using the Wizard® Genomic DNA Purification Kit (PROMEGA, USA). The whole genome sequencing was carried out at the Mutualized Microbiology Platform (P2M) of the Pasteur International Bioresources network (PIBnet) of the Institut Pasteur, Paris, France, using the Nextera XT DNA sample preparation kit (Illumina, USA) for 2 × 150-bp paired-end sequencing as per the manufacturer’s instructions. All sequenced paired-ends reads were clipped and trimmed with AlienTrimme [18], corrected with Musket [19], merged (if needed) with FLASH [20], and subjected to a digital normalization procedure with khmer [21]. For each sample, remaining processed reads were assembled and scaffolded with SPAdes [22]. Genomes were analyzed using the anti-SMASH 3.0 web server (http://antismash.secondarymetabolites.org) and the biosynthetic gene clusters were identified. The newly isolated *Aequorivita* sp. strain was deposited in the Collection of Institut Pasteur (CIP), France, under accession number CIP 111184. Moreover, the three genomes were deposited in European Nucleotide Archive (ENA) with codes: *Aequorivita* sp. GCA_900489465.1, *A. lipolytica* GCA_900489485.1 and *A. antarctica* GCA_900489835.1.

### 2.5 Cultivation and extract preparation

A single colony of a bacterial isolate was used to inoculate 3 mL of liquid Marine Broth (MB, Difco™) medium in sterile bacteriological tubes. After 48 h of incubation at 20 °C at 200 rpm, the culture was used to inoculate 125 mL of MB medium in a 500-mL flask, at an initial cell concentration of 0.01 OD_600_/mL. The flasks were incubated up to five days at 20 °C at 200 rpm. The cultures were then centrifuged at 6000 rpm at 4 °C for 30 min. Then, the exhausted culture broth and the pellet were collected and subjected to extraction with ethyl acetate separately, yielding the extracellular and intracellular extracts, respectively. Briefly, the exhausted culture broths (125 mL) were subjected to organic extraction using ethyl acetate (3 x 125 mL, ratio 1:1) in a 500 mL separatory funnel. The organic phase collected was evaporated to dryness using a Laborota 4000 rotary evaporator (Heidolph, Schwabach, Germany) affording the extracellular extract. The pellet was mechanically disrupted using an Ultraturrax for 5 min at 10,000 rpm and then extracted by ethyl acetate as described for the exhausted broth, providing the intracellular extract. The dried extracts were weighed, dissolved in 100% DMSO at the final concentration of 50 mg/mL and stored at -20 °C.

### 2.6 Pathogenic bacteria growth conditions

The following multidrug-resistant strains of human pathogens were used in this work: *Pseudomonas aeruginosa* DSM 50071, *Staphylococcus aureus* DSM 346, methicillin-resistant *Staphylococcus aureus* (*MRSA*) DSM 18827, *Acinetobacter baumannii* DSM 300007 *Klebsiella pneumonia* DSM 30104 (Leibniz Institute DSMZ-German Collection of Microorganisms and Cell Cultures, Braunschweig, Germany). The strains were routinely grown at 37 °C in Luria Bertani medium (10 g/L Tryptone, 5 g/L Yeast extract, 10 g/L NaCl) at 200 rpm.

### 2.7 Antimicrobial activity

To evaluate the antimicrobial potential of the *Aequorivita* sp. extracts, samples were placed into a 96-well microtiter plate at initial concentration of 1000 μg/mL. Then it was serially diluted using LB medium (2-fold dilution). The final extracts concentration was 500, 250, 120, 60, 30, 15 μg/mL. A single colony of selected pathogen strains, *P. aeruginosa* DSM 50071, *S. aureus* DSM 346, *MRSA* DSM 18827, *A. baumannii* DSM 300007 and *K. pneumonia*e DSM 30104 was used to inoculate 3 mL of liquid LB medium in sterile bacteriological tube. DMSO was used as negative control and 25 μM Doxycycline for *A. baumannii* DSM, 6 μM Chloramphenicol + 25 μM Penicillin for MRSA, 50 μM Chloramphenicol for *P. aeruginosa* and *K. pneumonia*e as positive control. After 6-8 h of incubation, growth was measured by monitoring the absorbance at 600 nm and about 0.04 OD/mL in 100 μL were dispensed in each well of the prepared plate reaching a final volume of 200 μL. Plates were incubated at 37 °C for 24 h and growth was measured with a Tecan plate reader by monitoring the absorbance at 600 nm. Values of bioactivity are reported in IC_50_, the half maximal inhibitory concentration, which is a measure of the potency of a substance in inhibiting a specific biological or biochemical function. In this case IC_50_ is used to describe the antimicrobial effect of a crude extract.

### 2.8 Anthelmintic activity

In order to test the effect of the crude extracts on *C. elegans* Wild-type (WT) Bristol N2 obtained from the *Caenorhabditis* Genetic Centre (CGC), a liquid toxicity assay has been set-up [23]. The assay was performed in 24-well plates. Each well contained a 400 μL solution of M9 buffer, 5 μg/mL cholesterol, and *Escherichia coli* OP50 at the concentration of 0.5 OD/mL as nutrient source [24]. The intracellular extract of *Aequorivita* sp. was then added at different concentrations, 500, 250, 120, 60, 30, 15 μg/mL to each well. *C. elegans* was synchronized by bleaching treatment [25], and 30–40 L4 worms were transferred to each well and incubated at 20 °C up to seven days. The wells were scored for living worms every 24 h. A worm was considered dead when it no longer responded to touch. For statistical purposes, 3 replicates per trial were carried out with a unique egg preparation. 1% (v/v) DMSO was used as negative control.

### 2.9 Cell viability assay (MTS assay)

The assay to determine cell survival was conducted by using HFFF2 (ECACC 86031405) cells as target and the MTS (3-(4,5-Dimethylthiazol-2-yl)-5-(3-carboxymethoxyphenyl)-2-(4-sulfophenyl)-2H-tetrazolium) as tetrazolium compound to determine the number of viable cells. Cells were seeded at 7.5 × 10^3^ cells/well in a sterile 96-well microtiter plate and allowed to adhere overnight by growing at 37 °C and 5% CO_2_. The various concentrations of *Aequorivita* sp. intracellular extracts (30, 62, 125, 250, 500 μg/mL) were added. DMSO, the solvent used to dissolve the extracts served as negative control. Three replicate wells were used per concentration. The treated cells were then incubated for 24 h at 37 °C and 5% CO_2_. After incubation, the medium was removed and a mixture consisting of 100 μl of fresh medium and 20 μL of reagent (MTS:Phenazine MethoSulfate 19:1) was added in each well. The plate was again incubated for 1 h at 37 °C and 5% CO_2_ in the dark to allow the reduction of MTS into a colored formazan product by living cells. In order to quantify the amount of formazan produced, directly proportional to the number of living cells after extracts treatment, absorbance was measured at 490 nm, using an ELISA plate reader.

## 3 Results

### 3.1 Isolation of Antarctic microorganisms using MCC

MCCs were used to isolate Antarctic bacteria from shallow water sediments. Two different conditions were used: one minimal, containing the Antarctic sediments as only nutrient source and the other richer, composed by sediments, peptone, yeast extract and sea salt were tested. After 10 days at 7 °C, the first colonies appeared, but the chip was kept in incubation up to 45 days, allowing the formation of slow-growing strains (Fig. 1). A total of 19 colonies were visualized by microscope and collected. The minimal and richer conditions showed similar numbers of colonies, 9 and 10 colonies respectively, and genera distribution indicating that natural sediments could contain a sufficient amount of nutrients to allow the microbial growth.

### 3.2 Identification of bacterial isolates

**Fig. 2:**
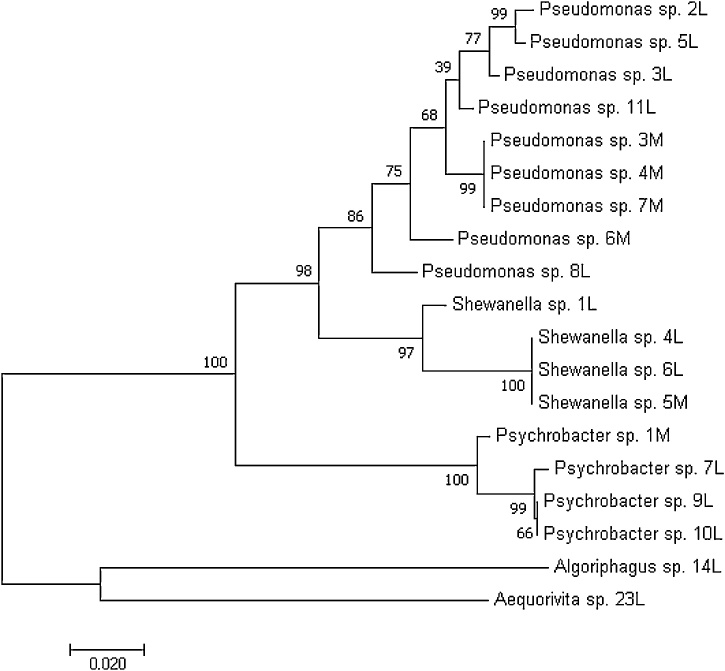
Evolutionary relationships of taxa and showing the placement of *Aequorivita* sp. (23 L) isolate. The evolutionary history was inferred using the Neighbor-Joining method. The optimal tree with the sum of branch length = 0.58717318 is shown. The percentage of replicate trees in which the associated taxa clustered together in the bootstrap test (1000 replicates) are shown next to the branches. The tree is drawn to scale, with branch lengths in the same units as those of the evolutionary distances used to infer the phylogenetic tree. The evolutionary distances were computed using the Maximum Composite Likelihood method and are in the units of the number of base substitutions per site. The analysis involved 19 nucleotide sequences.

The molecular analysis showed a prevalence of *Pseudomonas* sp. (9 strains), an equal number of *Shewanella* sp. and *Psychrobacter* sp. (4 strains), one *Algoriphagus* sp. and one *Aequorivita* sp. This last strain, showing 98% of identity with *Aequorivita antarctica* and 96% of identity with *Aequorivita sublithincola,* was obtained from the minimal condition and selected for further experiments because of the lack of previous studies about the bioactivity displayed by *Aequorivita* genus. A phylogenetic tree was built and the results are shown in Fig. 2.

### 3.3 Genomics of *Aequorivita* sp

Due to the lack of information on the genus *Aequorivita*, the whole genome’s of *Aequorivita* sp., *A. lipolytica* CIP 107455^T^ and *A. antarctica* CIP 107457^T^ strains were sequenced and subjected to a genome mining approach in order to identify the presence of biosynthetic gene clusters (BGCs). The whole genome of *Aequorivita* sp. has a total length of 3.5 Mb and was deposited in the European Nucleotide Archive (ENA). The antibiotics and Secondary Metabolite Analysis Shell (anti-SMASH) has served as a comprehensive web server and a stand-alone tool for the automatic genomic identification and analysis of BGCs, facilitating rapid genome mining of a wide range of bacterial and fungal strains. Using the anti-SMASH 3.0 web server, it is possible to compare identified BGCs with those encoding the biosynthetic pathways for known compounds from the “Minimum Information about a Biosynthetic Gene cluster” (MIBiG) community project (http://mibig.secondarymetabolites.org/) [26]. The gene cluster identification algorithms in tools such as anti-SMASH is based on the detection of one or more conserved protein domains using curated models; certain protein domains or domain combinations are indicative of biochemical functions that are specific to certain biosynthetic pathways; hence they can be used as “signatures” to identify certain classes of BGCs [27]. The cluster arylpolyene-resorcinol, similar to the flexirubin biosynthetic gene cluster and the arylpolyenes (APE) biosynthetic gene cluster, were found in the genome of *Aequorivita* sp. as well as in the genome of the *A. antarctica* CIP 107457^T^ and *A. lipolytica* CIP 107455^T^ (Table 1). Different saccharide clusters were detected in all *Aequorivita* strains, mainly a cluster similar to the capsular polysaccharide biosynthetic gene. Furthermore, in the genome of *Aequorivita* sp. strain a gene with 69% of identity with the Type III polyketide synthase of *Zobellia galactanivorans* was detected in the gene cluster T3pks-arylpolyene (data not shown). An NRP/Polyketide cluster was detected in the *A. lipolytica* CIP 107455^T^ strain (Table 1).

**Table 1:** Biosynthetic gene clusters (BGCs) in the *Aequorivita* sp. whole genome compared with two *Aequorivita* type strains: *A. antarctica* CIP 107457^T^ and *A. lypolitica* CIP 107455^T^) using the anti-SMASH 3.0 web server (http://antismash.secondarymetabolites.org).

Gene Cluster	Type	MIBiG BGC-ID	Most similar known cluster	*Aequorivita* sp.	*Aequorivita antarctica* CIP 107,457 T	*Aequorivita lipolytica* CIP 107,455 T
Cf_saccharide	Saccharide	BGC0000733_c1	Capsular polysaccharide biosynthetic gene cluster	X	X	X
Cf_saccharide	Saccharide	BGC0000773_c1	Lipopolysaccharide biosynthetic gene cluster; O-antigen biosynthetic gene cluster	X	–	–
Arylpolyene-Resorcinol	Polyketide	BGC0000838_c1	Flexirubin biosynthetic gene cluster; Aryl polyene (APE) biosynthetic gene cluster	X	X	X
Cf_saccharide	Saccharide	BGC0000766_c1	Exopolysaccharide biosynthetic gene cluster	–	X	X
Cf_saccharide	Saccharide	BGC0000781_c1	O-antigen biosynthetic gene cluster	X	–	–
Cf_saccharide	Saccharide	BGC0000791_c1	O-antigen biosynthetic gene cluster; Lipopolysaccharide biosynthetic gene cluster; Exopolysaccharide biosynthetic gene cluster	X	–	–
Cf_saccharide	Saccharide	BGC0000799_c1	Colanic acid biosynthetic gene cluster	–	X	–
Cf_saccharide	NRP / Polyketide	BGC0000960_c1	Azinomycin B biosynthetic gene cluster	–	–	X

### 3.4 Evaluation of the antimicrobial activity of *Aequorivita* sp.

In order to test the capability of the new isolate to produce antimicrobial compounds, intracellular and extracellular extracts from the *Aequorivita* sp. culture were generated. The bacterium was grown for five days in MB, producing a very strong yellow pigment. Results showed in Table 2 demonstrated that extracellular extract is totally inactive, while the intracellular extract shows an antimicrobial effect against three out of four-target pathogens. In particular, the most promising activity was detected against MRSA (IC_50_ value of 125 μg/mL), the only Gram-negative tested. Increasing the extract concentration, an inhibition effect was visible against *A. baumannii* (IC_50_ value of 250 μg/mL) and *P. aeruginosa* (IC_50_ value of 500 μg/mL). No effect was detected against *K. pneumoniae.* The extracts at 60, 30 and 15 μg/mL are not shown in the figures and tables.

**Table 2:** Antimicrobial activity of *Aequorivita* sp. intracellular and extracellular extracts against a panel of MDR bacteria. Positive control has been performed using antibiotics: Doxycycline, Chloramphenicol and Penicillin. Bacterial growth was measured in OD_600_/mL.

	*IC_50_ value*		
*Strain*	Intracellular	Extracellular	Positive control
*P. aeruginosa*	*500 μg/mL*	No inhibition	50 μM Chl
*MRSA*	*120 μg/mL*	No inhibition	6 μM Chl + 25 μM Pe
*A. baumannii*	*250 μg/mL*	No inhibition	25 μM Dox
*K. pneumoniae*	No inhibition	No inhibition	50 μM Chl

### 3.5 Anthelmintic activity of *Aequorivita* sp. crude extracts

**Fig. 3:**
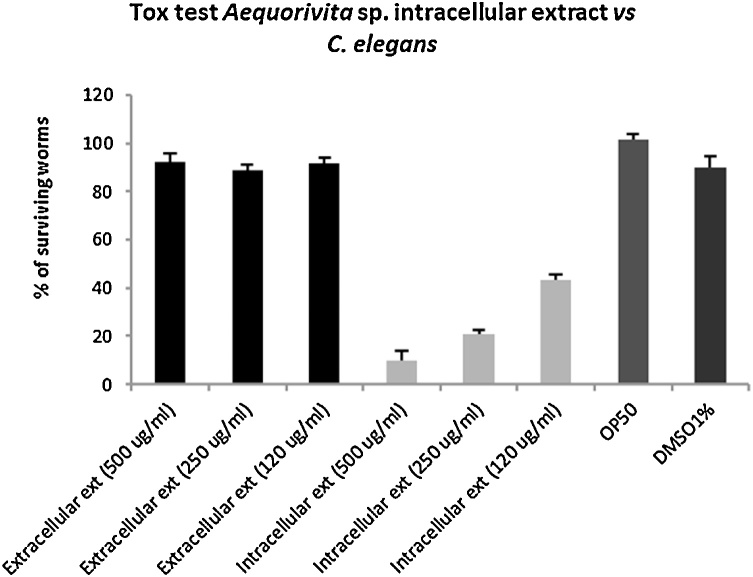
Anthelmintic activity of *Aequorivita* sp. extracellular (Black bars) and intracellular extracts (Light grey bars). Activity is expressed as the percentage of surviving worms after 3 days of incubation in the presence of extracts. DMSO plus worms and OP50 plus worms were used as controls.

Both intra and extracellular crude extracts were also tested for their ability to kill nematodes using *C. elegans* as model system. Again, only the intracellular extract had significant *in vitro* anthelmintic effect after 3 days of incubation. The intracellular extract was able to kill 90% of worms at the concentration of 500 μg/mL, 80% and 60% at respective concentrations of 250 μg/mL 120 μg/mL as showed in Fig. 3. The extracts at 60, 30 and 15 μg/mL are not shown in the figures and tables.

### 3.6 Evaluation of *Aequorivita* sp. extract toxicity

**Fig. 4:**
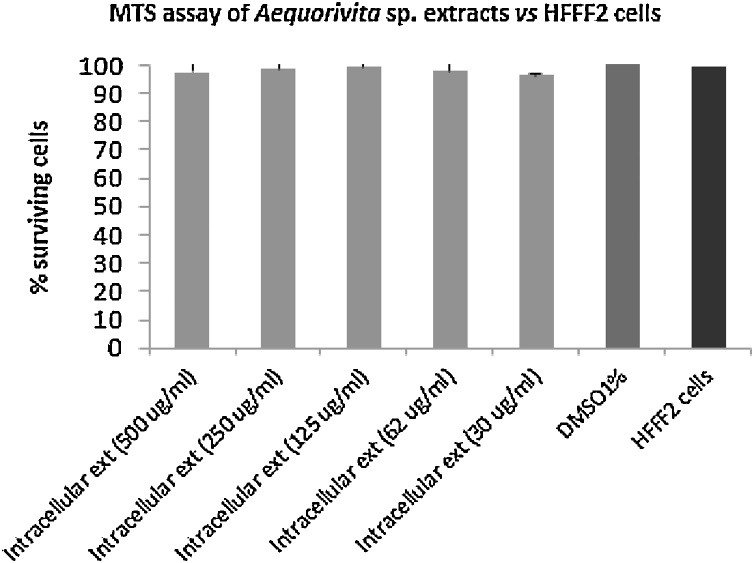
Cell viability assay using HFFF2 cells in presence of *Aequorivita* sp. intracellular extract (Light grey bars). Values are reported as percentage of surviving cells. DMSO plus HFFF2 cells and HFFF2 alone were used as controls.

Cell viability assays were carried out by using human fibroblastic cells HFFF2 (ECACC 86031405) that represent a model system of skin cells. The value of percentage of viable cells after the treatment was obtained by normalizing the value of absorbance in each well with the correspondent value of absorbance of DMSO, at the same concentration. Only the intracellular extract was evaluated for its toxicity. Results demonstrated that the crude extract has no toxic effect against HFFF2 cells (Fig. 4) even at the highest tested concentration 500 μg/mL, which is the concentration that always showed antimicrobial and anthelmintic activity. Results are shown as percentage of viable cells after the treatment at different concentration of sample.

## 4 Discussion

Microbes have been a major source of natural antibiotics playing a very significant role in historical and modern antibiotic drug discovery [28]. However, these efforts are seriously hampered by their limited cultivability in artificial laboratory conditions because most of them don’t grow when extrapolated from their natural environment [29]. Hence different strategies are mandatory for successful isolation and cultivation of strains from new, untapped environments. In this work, we have isolated several microorganisms from an extreme environment, Antarctica, by using a Miniaturized Culture Chip (MCC) [7]. The main idea of MCC is the simulation of natural environment, by laying the chip directly on natural sediments that become the only substrate for the growth of microorganisms. By the diffusion of nutrients obtained from sediments and by stimuli received from the bacterial community present in it, microbes can grow on the top of the chip as microcolonies. They are partially protected by the overgrowth of the neighbouring microbes but available for imaging and recovery. A similar idea forms the basis of the iCHIP, developed by Lewis and Epstein [30], that is composed by microwells and semipermeable membranes. The use of iCHIP allowed Ling et al., to isolate a new β-proteobacteria provisionally named *Eleftheria terrae* able to produce a new antibiotic molecule, teixobactin [31]. The choice for studying Antarctic sediments as a source of new strains is due to the fact that Antarctica is a unique habitat. It is one of the most hostile places on Earth, characterized by low temperatures and other extreme traits, such as long light/dark period and poor nutrient levels. Such extreme conditions would be expected to shape the evolution and adaptation of both primary and secondary metabolism of the Antarctic bacteria, to lead to the production of chemical molecules with unique functions [32]. Moreover, a survey conducted by the international Census of Marine Life demonstrated the high biodiversity of this extreme habitat and the marine environment around it [33]. In this study, the application of the MCC to Antarctic sediments led to the isolation of different genera of bacteria. Two nutritional conditions (A minimal; B richer) were applied in order to see some changes in the microbial community. In particular, from the chip, bacteria belonging to the genus *Pseudomonas* sp., *Shewanella* sp. and *Psychrobacter* sp. together with an *Algoriphagus* sp. and an *Aequorivita* strain have been retrieved. Genera like *Pseudomonas*, *Shewanella* and *Psychrobacter* include many different species and are commonly retrieved from a wide range of marine environments meaning that their genetic and metabolic potential is highly versatile. One example is represented by *Shewanella violacea,* a violet gram-negative bacterium isolated from marine sediment in the Ryukyu Trench at a depth of 5110 m [34]. In this work, a similar number of colonies were observed on the two nutritional conditions without any significant variation in the strain distribution. This could indicate that bacteria used natural sediments as primary nutrient source for the growth, highlighting the importance of the reproduced microenvironment for bacterial survival. The diffusion of nutrients from sediments, the communication among microorganisms up and down the MCC, the protection of slower growing strains by overgrowth of more aggressive or rapidly growing strains by the MCC wells, could be all be important factors that allowed the cultivation of microorganisms. As confirmed by Pulschen et al., who was able to isolate several previously uncultured Antarctic strains, long incubation period and low nutritional media are still useful and effective approach for the isolation of rare/novel microorganisms [35]. Several strains belonging to the genus *Aequorivita* have been isolated from Antarctica (sea water, ice, algae, stones) but few studies about these bacteria were performed such as taxonomy, nutrients requirements, growth conditions, DNA G + C content, fatty acid composition [15]. The lack of any previous study evaluating the bioactive potential of this genus makes this strain a good candidate for further studies. The whole genome sequencing of *Aequorivita* sp. and its analysis by anti-SMASH indicated the presence of several BGCs. BGCs have been described for hundreds of bacterial metabolites and even though they can be accurately identified and quantified, the question still remains, which of these are most likely to encode the production of potent antimicrobials. The most prominent family of BGCs already described includes two subfamilies distributed throughout the Proteobacteria; their products are aryl polyenes (APEs) [36] also found in *Aequorivita* sp. and in other strains. Although these clusters are widely divergent in sequence, their small molecules are remarkably conserved, suggesting the important role that these compounds play in Gram-negative cell biology. The APEs are structurally similar to flexirubin, a pigment that was previously isolated from *Flexibacter elegans* [37]. A role for APEs in protecting Gram-negative bacteria against oxidative stress makes them analogous to the chemically similar Gram-positive carotenoids [36] although they are biosynthetically distinct. The strong yellow/orange *Aequorivita* sp. pigmentation could suggest the production of these molecules, so future studies, aimed at the evaluation/selection of the best growth condition for the expression of antioxidant compounds, will be performed. In addition, numerous gene clusters encoding genes for saccharides were found. Cell-associated saccharides, such as lipopolysaccharides and capsular polysaccharides are known to play key roles in microbe-host and microbe-microbe interactions, while diffusible saccharides have a range of biological activities, most notably antibacterial [38,39]. Non-ribosomal peptides (NRPs) are the major multi-modular enzyme complex which synthesizes secondary metabolites in bacteria and fungi. In the genome of *Aequorivita* sp. a gene with 69% of identity with the Type III polyketide synthase of *Zobellia galactanivorans* was detected in the gene cluster T3pks-arylpolyene (data not shown), indicating a possible role of this cluster in the production of polyketides. The lack of any data on bioactive potential of *Aequorivita* genus motivated for the cultivation of the isolated *Aequorivita* sp. strain and the evaluation of antimicrobial and anthelmintic activity of its extracellular and intracellular extract. Analysis of the antimicrobial potential of this bacterium revealed a promising inhibitory activity against some pathogenic MDR bacteria. In particular, the intracellular crude extract of *Aequorivita* sp. (obtained by mechanical disruption of bacterial cells followed by organic extraction) showed promising antimicrobial effect against MRSA, the causative agents of serious hospital infections. Vancomycin and linezolid have become the only drugs to counteract the MRSA infections, however, treatment failures, adverse side effects and the early developed resistance highlight the necessity of alternative therapies [40]. The lack of new antibiotic molecules entering the market and the overuse/misuse of the existing antibiotics are generating an alarming situation for human health. This phenomenon involves not only infections caused by MDR bacteria but also helminth infections. Millions of people are affected by helminth infections each year and new anthelmintic agents are urgently needed to treat and control these diseases [41]. In this study, we have shown *Aequorivita* sp. intracellular extract to be active, without toxic effects on HFFF2 cells, towards the nematode *C. elegans,* a model system used to in screening of anthelmintic drugs. Considering that the bioactivity was evaluated using crude extracts, the antimicrobial activity may increase after several purification step of the extract. The fact that both antimicrobial and anthelmintic activities are expressed by the intracellular extract is not surprising. Most of antimicrobials are expressed outside the cell for cell defense but the intracellular nature of some antimicrobial compounds is not unusual, although their ecological role is still not clear [42,43]. They could be involved in intracellular signalling or have a defensive role [44]. A bioassay-guided purification strategy should lead to the identification of natural compounds responsible for the bioactivity and further studies will be necessary to fully understand the mechanisms of action of the identified molecules. In particular, the scale-up of the culture will allow obtaining a sufficient amount of crude intracellular extract for the putification, by using Solid Phase Extraction column and HPLC. Finally, High Resolution Mass Spectrometry and Nuclear Magnetic Resonance will be used to identify the structure of the bioactive compounds. Moreover, the implementation of genomics techniques could guide the cultivation of this strain in different conditions inducing the expression of the BGCs present in the genome of *Aequorivita* sp.

## 5 Conclusions

In this work a novel isolation method using a Miniaturized Culture Chip (MCC) allowed the isolation of an unstudied Antarctic bacterium belonging to the genus *Aequorivita*. The analysis of the bioactive potential of the intracellular and extracellular extracts of this strain showed a promising antimicrobial and anthelmintic activity. This is the first multi-approach study on the genus *Aequorivita* involving the isolation by MCC, the analysis of BGCs, and the evaluation of the bioactivity.

## Conflicts of interest

The authors declare that there are no conflicts of interest.

## Funding

This research is partially funded by the MarPipe project: Improving the flow in the pipeline of the next generation of marine biodiscovery scientists, funded through the European Commission H2020-MSCA-ITN-ETN scheme, GA721421.
